# Birth defects reporting and the use of dydrogesterone: a disproportionality analysis from the World Health Organization pharmacovigilance database (VigiBase)

**DOI:** 10.1093/hropen/hoae072

**Published:** 2025-01-02

**Authors:** Alexandra Henry, Pietro Santulli, Mathilde Bourdon, Chloé Maignien, Charles Chapron, Jean-Marc Treluyer, Jean Guibourdenche, Laurent Chouchana

**Affiliations:** Regional Center of Pharmacovigilance, Pharmacology Department, Cochin Hospital, AP-HP.Centre—Université Paris Cité, Paris, France; UMR1343, « Pharmacologie et évaluation des thérapeutiques chez l’enfant et la femme enceinte », INSERM, Université Paris Cité, Paris, France; Faculté de Santé, Université Paris Cité, Paris, France; Department of Gynaecology Obstetrics II and Reproductive Medicine, Cochin Hospital, AP-HP.Centre—Université Paris Cité, Paris, France; Department 3I « Infection, Immunité et inflammation », Institut Cochin, INSERM U1016, Paris, France; Faculté de Santé, Université Paris Cité, Paris, France; Department of Gynaecology Obstetrics II and Reproductive Medicine, Cochin Hospital, AP-HP.Centre—Université Paris Cité, Paris, France; Department 3I « Infection, Immunité et inflammation », Institut Cochin, INSERM U1016, Paris, France; Faculté de Santé, Université Paris Cité, Paris, France; Department of Gynaecology Obstetrics II and Reproductive Medicine, Cochin Hospital, AP-HP.Centre—Université Paris Cité, Paris, France; Faculté de Santé, Université Paris Cité, Paris, France; Department of Gynaecology Obstetrics II and Reproductive Medicine, Cochin Hospital, AP-HP.Centre—Université Paris Cité, Paris, France; Department 3I « Infection, Immunité et inflammation », Institut Cochin, INSERM U1016, Paris, France; Regional Center of Pharmacovigilance, Pharmacology Department, Cochin Hospital, AP-HP.Centre—Université Paris Cité, Paris, France; UMR1343, « Pharmacologie et évaluation des thérapeutiques chez l’enfant et la femme enceinte », INSERM, Université Paris Cité, Paris, France; Faculté de Santé, Université Paris Cité, Paris, France; Department of Hormonology, Cochin Hospital, AP-HP.Centre—Université Paris Cité, Paris, France; Regional Center of Pharmacovigilance, Pharmacology Department, Cochin Hospital, AP-HP.Centre—Université Paris Cité, Paris, France; UMR1343, « Pharmacologie et évaluation des thérapeutiques chez l’enfant et la femme enceinte », INSERM, Université Paris Cité, Paris, France

**Keywords:** birth defect, dydrogesterone, assisted reproductive technology, ART safety, congenital heart defect, hypospadias, drug safety

## Abstract

**STUDY QUESTION:**

Is there an association between dydrogesterone exposure during early pregnancy and the reporting of birth defects?

**SUMMARY ANSWER:**

This observational analysis based on global safety data showed an increased reporting of birth defects, mainly hypospadias and congenital heart defects (CHD), in pregnancies exposed to dydrogesterone, especially when comparing to progesterone.

**WHAT IS KNOWN ALREADY:**

Intravaginal administration of progesterone is the standard of care to overcome luteal phase progesterone deficiency induced by ovarian stimulation in ART. In recent years, randomized controlled clinical trials demonstrated that oral dydrogesterone was non-inferior for pregnancy rate at 12 weeks of gestation and could be an alternative to micronized vaginal progesterone. Safety profiles in both mother and child were similar. However, concerns have been raised regarding an association between dydrogesterone usage during early pregnancy and CHD in offspring.

**STUDY DESIGN, SIZE, DURATION:**

We performed a disproportionality analysis, also called case–non-case study, similar in concept to case–control studies, using the WHO global safety database, VigiBase. The study cohort consisted of individual pregnancy-related safety reports, using the *ad hoc* standardized query (SMQ ‘Pregnancy and neonatal topics’). Cases of birth defects consisted of safety reports containing terms related to the ‘congenital, familial and genetic disorders’ System Organ Class from the Medical Dictionary for Regulatory Activities. Non-cases consisted of safety reports containing any other adverse event, in pregnancy-related safety reports.

**PARTICIPANTS/MATERIALS, SETTING, METHODS:**

Considering reports since database inception to 31 December 2021, we first compared the reporting of birth defects with dydrogesterone to that of any other drug on the database, then to any other drug used for ART. Secondly, we performed a comparison on the reporting of birth defects for dydrogesterone with progesterone. Results are presented as reporting odds ratio (ROR) and their 95% CI. For each comparison, two sensitivity analyses were performed. Finally, a case-by-case review was performed to further characterize major birth defects and sort anomalies according to classification of EUROCAT.

**MAIN RESULTS AND THE ROLE OF CHANCE:**

Study cohort consisted of 362 183 safety reports in pregnant women, among which 3101 reports were related to the use of drugs for ART, including 145 with dydrogesterone and 1222 with progesterone. Of these, 374 (12.1%) were cases of birth defects: 60 with dydrogesterone and 141 with progesterone, including 48 and 92 cases compatible with major birth defect cases according to EUROCAT classification, respectively. Major birth defects reported with dydrogesterone were mainly genital defects such as hypospadias and CHD. A significantly higher disproportionate reporting of birth defects was found with dydrogesterone when compared to any other drug (ROR 5.4, 95% CI [3.9–7.5]), to any other ART drug (ROR 6.0, 95% CI [4.2–8.5]), and to progesterone (ROR 5.4, 95% CI [3.7–7.9]). Sensitivity analyses found consistent results.

**LIMITATIONS, REASONS FOR CAUTION:**

First, under-reporting, being inherent to pharmacovigilance systems, impedes the measurement of the incidence of adverse drug reactions and can limit the sensitivity of signal detection. Second, drug causality, not being the same for all cases, is challenging for such events and requires further assessment. However, sensitivity analyses showed consistent results.

**WIDER IMPLICATIONS OF THE FINDINGS:**

This possible safety signal emphasizes the need for further investigation regarding the fetal safety profile of dydrogesterone.

**STUDY FUNDING/COMPETING INTEREST(S):**

No funding was received for this study. None of the authors have any financial or personal relationships with other people or organizations that could influence the design, conduct, or reporting of this work. M.B., C.C, C.M and P.S have received institutional funding from Merck, Ferring, Theramex, Gedeon Richter, and Besins. M.B. has received personal honoraria from Merck, Ferring, Gedeon Richter, Theramex, IBSA, and Organon for lectures, presentations, or educational events, and has received non-monetary support for attending meetings from Ferring and Gedeon Richter. C.C has received personal honoraria from Merck, Besins, Gedeon Richter, and Theramex, and non-monetary support for attending meetings from Besins, Gedeon Richter, and Merck. He is the Founder and Past-President of the Society for Endometriosis and Uterine Disorders (SEUD), an unpaid role. C.M. has received personal consulting fees from Gedeon Richter and Ferring and honoraria from Merck Serono, Ferring, Besins, IBSA, and Organon for lectures or educational events. She has received non-monetary support for attending meetings from Ferring, Besins, and Gedeon Richter. P.S. has received personal honoraria from Merck, Ferring, Besins, Gedeon Richter, Theramex, IBSA, and General Electrics Medical Systems for lectures and educational events. He has also received non-monetary support for attending meetings from Merck, Ferring, Besins, Gedeon Richter, Theramex, and IBSA. He is a Board Member of SEUD and serves on the Editorial Boards of RBMO and GOF, all unpaid roles. J.G. has received institutional funding from Pregnomic and DiaSorin. He has received personal honoraria from Elsevier and Merck for lectures and educational events. He is a Board Member of ABA and SFE, an unpaid position. L.C., J.-M.T., and A.H. declare no conflicts of interest.

**TRIAL REGISTRATION NUMBER:**

N/A.

WHAT DOES THIS MEAN FOR PATIENTS? This study looks at whether the side effects reported with dydrogesterone, a drug used to prevent early miscarriage notably in assisted reproductive technology, show a significant amount of birth defects. This study looked back at the data reported by patients or healthcare professionals to the pharmacovigilance systems at a global level, using the safety database managed by the World Health Organization. It found that birth defects are statistically more reported with dydrogesterone than with other drugs used in assisted reproductive technology, including progesterone. The results do need to be treated with some caution because the study methodology cannot establish causation and further research is needed.

## Introduction

It has been estimated that about 190 million individuals worldwide live with infertility (The Lancet Global Health, 2022). As inadequate progesterone serum levels during the luteal phase of the menstrual cycle and early pregnancy can cause a miscarriage, progestins are commonly used to prevent early miscarriage and preterm labor (Devall *et al.*, 2021). In ART, intravaginal administration of micronized vaginal progesterone is the standard of care to overcome luteal phase deficiency induced by ovarian stimulation (Ovarian Stimulation TEGGO *et al.*, 2020). In recent years, two randomized controlled clinical trials demonstrated that oral dydrogesterone, a man-made progesterone derivative, was non-inferior for pregnancy rate at 12 weeks of gestation and could be an alternative (Tournaye *et al.*, 2017; Griesinger *et al.*, 2018). In these studies, the safety profiles in both mother and child appeared comparable to micronized vaginal progesterone. Overall, these findings provide grounds for the routine use of oral dydrogesterone for luteal phase support in ART (Griesinger *et al.*, 2020). Dydrogesterone is now widely used to prevent miscarriage, and exposure in early pregnancy may be common in the overall population in certain regions (Walch *et al.*, 2005; Katalinic *et al.*, 2022).

Although the safety profile from clinical trials is reassuring, concerns have been raised regarding an association between dydrogesterone exposure during early pregnancy and congenital heart defects (CHD) in the offspring (Zaqout *et al.*, 2015). A recent study from China also identified an association with birth defects and stillbirth (Li *et al.*, 2024). Hence, taking advantage of the WHO global safety database, we sought to provide further data regarding the association between dydrogesterone use and the reporting of birth defects, including a case-by-case analysis of the reported birth defects.

## Materials and methods

### Data source

We conducted a retrospective, observational, disproportionality analysis, using a case–non-case approach, of spontaneous reports from the world’s largest database of individual case safety reports, VigiBase (https://who-umc.org/vigibase/). This database, which has been implemented since 1968, following the thalidomide disaster, is part of the WHO Program for International Drug Monitoring. To date, it comprises about 29 million reports of suspected adverse drug reactions, spontaneously reported by healthcare professionals or patients and collected by national drug authorities of 150 countries. Each safety report is related to a single patient exposed to one or more drugs suspected to cause an adverse drug reaction. For each report, the database contains information including suspected and concomitant drugs, medical history, the type of adverse event, including adverse pregnancy outcomes in cases related to pregnant women, coded using terms from the Medical Dictionary for Regulatory Activities (MedDRA, https://www.meddra.org/), patient demographics and reporter occupation (healthcare professionals or patients).

### Study cohort and case identification

For this study, the patient cohort consisted of individual case safety reports regarding pregnant women. To conduct signal detection, a patient cohort including pregnancy-related reports was constituted using the Standardized MedDRA Query ‘Pregnancy and neonatal topics’ (Wisniewski *et al.*, 2016). In the cohort, safety reports might concern the pregnant woman (between 18- and 44-year-old), the fetus (age reported as unknown), or the infant (age below 2 years), according to national coding guidelines for pregnancy-related safety reports. Cases of birth defects consisted of reports containing terms related to the ‘congenital, familial and genetic disorders’ System Organ Class (SOC) from MedDRA. Safety reports containing terms related to chromosomal or genetic abnormalities, genetic mitochondrial abnormalities, congenital infections, inborn errors of metabolism, and lysosomal storage disorders were excluded. Furthermore, case-by-case review was performed by a tandem of junior (A.H.) and senior (L.C.) clinical pharmacologists to (i) ascertain birth defect cases in safety reports, (ii) identify the presence of other teratogen agents (Supplementary Table S1), (iii) sort cases of major birth defects according to classification of the European network of population-based registries for the epidemiological surveillance of major congenital anomalies (EUROCAT, https://eu-rd-platform.jrc.ec.europa.eu/EUROCAT_en), and (iv) remove duplicate records. Non-cases were safety reports containing any other event. The study period was from database inception in 1968 to 31 December 2021.

### Exposure

Drug exposure of interest was dydrogesterone, progesterone, as well as any drug used for ART: GnRH antagonists (cetrorelix, ganirelix), gonadotropins for follicle stimulating and for ovulation induction, clomifene and letrozole. For drugs used both in ART and in a few non-ART indications such as letrozole, reports related to non-ART indications, or when indication was not known, were excluded from the study. GnRH agonists, although used in ART, were excluded from drug exposure of interest because of too many reports with non-ART indications.

### Statistical analysis

Disproportionality analysis, also called case–non-case study, is a statistical approach that is conceptually similar to a case–control study for pharmacovigilance purpose. It estimates whether there is a differential reporting regarding a specific adverse event in a group exposed to a specific drug, compared to reports of the same adverse event in a control group exposed to any other drug or to a pre-determined control drug. The association is expressed as a reporting odds ratio (ROR) and its 95% CI, which is similar in concept to the odds ratio reported in case–control studies. A lower boundary of 95% CI greater than one is deemed significant and expresses a disproportionate reporting of the adverse event of interest with the drug of interest (Faillie, 2019). In the absence of signal, there is an independent distribution of cases and non-cases (i.e. similar between reports exposed to the specific drug and those exposed to the control group).

To assess a signal of disproportionate reporting for birth defects with the use of dydrogesterone, we conducted a three-step analysis. First, we compared the raw frequency of birth defects reported with dydrogesterone to any other drug in study cohort. Second, to mitigate indication bias, we compared the reporting of birth defects with dydrogesterone to any other drug used for ART (Grundmark *et al.*, 2014). Third, we performed a head-to-head comparison for the reporting of birth defects between dydrogesterone and progesterone. By using active comparators, the analysis performance improves and has a better ability to detect true positives (Grundmark *et al.*, 2014). Finally, for each analysis, two sensitivity analyses were performed, restricted to (i) reports from 2001 to 2020, and (ii) reports originating from healthcare professionals. Statistical analyses were performed using Microsoft Excel software from Microsoft Corporation (Redmond, WA, USA).

### Ethics

VigiBase is managed by the Uppsala Monitoring Center (UMC, Uppsala, Sweden), an independent center for drug safety and scientific research for WHO. All safety reports are anonymized with no possibility to trace back patients’ or reporters’ personal information. Data were treated following EU and national legislation regarding the protection of personal data (e.g. the Data Protection Directive 95/46/EC and Regulation (EC) No 45/2001). Ethics approval for this study was waived. The study was approved by our local institutional review board (AAA-2023-09053).

This study followed the guideline for REporting of A Disproportionality Analysis for DrUg Safety Signal Detection Using Individual Case Safety Reports in PharmacoVigilance (READUS‐PV) (Fusaroli *et al.*, 2024).

## Results

### Cases description

The study cohort consisted of 362 183 safety reports in pregnant women, among which 3101 safety reports were related to the use of drugs for ART (of 50 653 reports overall with these drugs regardless of the indication), including 145 with dydrogesterone and 1222 with progesterone (Supplementary Fig. S1, Supplementary Table S2). Of these, query retrieved 470 possible cases of birth defects in reports with ART drugs, including 374 (12.1%) that were retained as cases of birth defects after case-by-case review: 60 birth defects cases were reported with dydrogesterone and 141 with progesterone. Birth defect cases were mostly reported from Europe (73%) and Asia (22%) for dydrogesterone, and from Europe (53%) and North America (33%) for progesterone (Table 1). The majority of cases were reported during the last decade by physicians. Median [interquartile range] maternal age was comparable between dydrogesterone (30 [25–33.5] years) and progesterone (32 [30.3–35.3] years). In all except 6 (4%) cases reported with progesterone, no other teratogenic drug was suspected in the onset of birth defect cases.

**Table 1.** Characteristics of birth defect cases reported with dydrogesterone, progesterone, and with any drugs used for ART within the WHO global safety database.

Reporting characteristics	Dydrogesterone	Progesterone	Any ART drug
	(n = 60)	(n = 141)	(n = 374)
**Continent of reporting**			
Europe	44 (73%)	75 (53)	214 (57%)
North America	1 (2%)	47 (33%)	89 (24%)
Asia	13 (22%)	15 (11%)	57 (15%)
Oceania	–	1 (1%)	11 (3%)
Africa	1 (2%)	2 (1%)	3 (1%)
Latin America	1 (2%)	1 (1%)	1 (0%)
**Type of reporter**			
Physician	49 (82%)	85 (60%)	239 (64%)
Unknown	4 (7%)	13 (9%)	67 (18%)
Consumer	7 (12%)	36 (26%)	58 (16%)
Other Health professional	–	14 (10%)	22 (6%)
Pharmacist	–	4 (3%)	6 (2%)
**Reporting year**			
Before year 1980	5 (8%)	1 (1%)	21 (6%)
Years 1980 to 1990	5 (8%)	1 (1%)	37 (10%)
Years 1991 to 2000	1 (2%)	7 (5%)	35 (9%)
Years 2001 to 2010	3 (5%)	31 (22%)	59 (16%)
Years 2011 to 2020	33 (55%)	92 (65%)	196 (52%)
Year 2021	13 (22%)	9 (6%)	27 (7%)
**Maternal age—years**	30 [25–33.5]	32 [30.25–35.25]	30 [26–33]
**Major birth defect** *			
Number of cases	48	92	^¶^
Number of anomalies reported	56	114	
**Concomitant teratogenic drug—number of cases** **			
None	60 (100%)	135 (95.7%)	^¶^
One	–	5 (3.5%)	
Two	–	1 (0.7%)	

Data are presented as N (%) or median [interquartile range].

WHO, world health organization.

* Major birth defects according to EUROCAT classification, guide version 1.5 (https://eu-rd-platform.jrc.ec.europa.eu/EUROCAT/data-collection/guidelines-for-data-registration_en). As one case may include more than one anomaly, number of anomalies is greater than the number of cases.

** Detailed list of teratogenic drugs: see Supplementary Table S1.

¶ Not performed for all assisted reproductive therapy.

### Birth defect analysis

After case-by-case review, 48 of 60 (80%) and 92 of 141 (65%) cases reported with dydrogesterone and progesterone respectively, met criteria for major birth defect according to EUROCAT classification. Regarding dydrogesterone, these 48 cases contained 56 major anomalies, consisting mainly in genital defects such as hypospadias (n = 18, 32%, including a cluster of 10 cases of hypospadias reported from United Arab Emirates in 2021) and CHD (n = 15, 27%) (Table 2). These anomalies were reported as unique in 41 of 48 cases (85%) and associated with another anomaly in 6 cases (13%), the remaining cases including multiple anomalies. More specifically for genital defects, 17 of 18 cases (94%) consisted of hypospadias standalone, while in one case (6%), it was associated with hydrocephalus. For CHD, anomalies were unique in all except 1 case of 15 (7%). Regarding progesterone, these 92 cases contained 114 major anomalies, consisting mainly of CHD (n = 37, 32%) and genital defects (n = 27, 24%). These anomalies were mostly (n = 72 of 92 cases, 78%) reported without any another anomaly. Focusing on genital defects, 24 of 27 cases (89%) reported as unique hypospadias, whereas for CHD, 10 of 37 cases (27%) reported at least another major anomaly (cardiac or other type).

**Table 2.** Type of major birth defect reported with dydrogesterone or progesterone, sorted by subgroup according to EUROCAT classification.

	Dydrogesterone	Progesterone	**Expected relative proportion of major anomaly subgroups** **
	n = 48	n = 92	
**All major anomalies** *	56	114	100%
**Nervous system**	4 (7%)	7 (6%)	11%
Neural tube defects	2	3	
Encephalocele	1	1	
Spina Bifida	1	1	
Neural tube defect (no precision)	0	1	
Hydrocephalus	2	1	
Severe microcephaly	0	3	
**Eye**	0 (0%)	0 (0%)	2%
**Ear, face and neck**	0 (0%)	0 (0%)	1%
**Congenital heart defects (CHD)**	15 (27%)	37 (32%)	33%
Severe CHD	2	6	
Double outlet right ventricle	1	1	
Transposition of great vessels	2	0	
Ventricular septal defect	2	15	
Atrial septal defect	1	12	
Atrioventricular septal defect	1	0	
Tetralogy of Fallot	1	1	
Tricuspid atresia and stenosis	1	0	
Pulmonary valve stenosis	1	0	
Aortic valve atresia/stenosis	1	0	
Hypoplastic left heart	0	2	
Coarctation of aorta	1	0	
Aortic atresia/interrupted aortic arch	1	0	
**Respiratory**	0 (0%)	0 (0%)	2%
**Oro-facial clefts**	3 (5%)	12 (11%)	6%
Cleft lip with or without palate	3	8	
Cleft palate	1	4	
**Digestive system**	6 (11%)	10 (9%)	8%
Oesophageal atresia with or without tracheo-oesophageal fistula	2	3	
Duodenal atresia or stenosis	0	2	
Atresia or stenosis of other parts of small intestine	0	1	
Ano-rectal atresia and stenosis	2	3	
Diaphragmatic hernia	2	1	
**Abdominal wall defects**	0 (0%)	3 (3%)	3%
Omphalocele	0	3	
**Urinary**	0 (0%)	9 (8%)	15%
Bilateral renal agenesis including Potter syndrome	0	7	
Multicystic renal dysplasia	0	1	
Congenital hydronephrosis	0	1	
**Genital**	18 (32%)	27 (24%)	10%
Hypospadias	18	27	
**Limb**	10 (18%)	8 (7%)	17%
Limb reduction defects	2	1	
Club foot—talipes equinovarus	5	4	
Polydactyly	0	1	
Syndactyly	3	2	
**Other anomalies/syndromes**	0 (0%)	1 (1%)	–
Congenital constriction bands/amniotic band	0	1	

Number of major birth defects, data are presented as N (%).

* As one case may include more than one anomaly, number of cases is superior to number of anomalies. Percentages are provided according to the total number of anomalies.

** Expected relative proportions of congenital anomaly subgroups were estimated using prevalence data from EUROCAT (data from 2001 to 2021, full registries, excluding genetic anomalies, dataset of 1 June 2023).

Genital defects appeared more disproportionately prevalent with dydrogesterone and progesterone when comparing with expected prevalence of these cases according to EUROCAT.

### Disproportionality analysis

In the primary analysis, a significant disproportionate reporting of birth defects was found with dydrogesterone when compared to any other drug in the study cohort (ROR 5.4, 95% CI [3.9–7.5]) and to any other ART drug (ROR 6.0, 95% CI [4.2–8.5]) (Table 3). In the head-to-head comparison, we found an increased reporting of birth defect with dydrogesterone compared to progesterone (ROR 5.4, 95% CI [3.7–7.9]). Results of the different sensitivity analyses, including a time-restricted analysis to the 2001–2020 period (i.e. that did not contain the reporting cluster of hypospadias in 2021), retrieved consistent results (Supplementary Table S3).

**Table 3.** Reporting and odds ratios of reporting of birth defects with dydrogesterone users within the WHO global safety database.

	Birth defect cases	Non cases	ROR (95% CI)
**Primary analysis**			
Dydrogesterone users	60	85	5.4 [3.9–7.5]
Any drug users	41 991	320 192	Ref
**Secondary analysis**			
Dydrogesterone users	60	85	6.0 [4.2–8.5]
Any other ART drug users	314	2642	Ref
**Head-to-head comparison** *			
Dydrogesterone users	60	85	5.4 [3.7–7.9]
Progesterone users	141	1081	Ref

ROR, reporting odds ratio.

* For detailed characteristics of all individual case safety reports suspected to be related to dydrogesterone or progesterone, see Supplementary Table S2.

## Discussion

Using the WHO global safety database, our analysis showed an important increased reporting of birth defects associated with dydrogesterone use during pregnancy, about five times higher when compared to progesterone use. Most reported birth defects with dydrogesterone were CHD and hypospadias, the latter appeared largely increased compared to expected proportion of major anomalies based on EUROCAT surveillance data.

Through disproportionality studies, the purpose of pharmacovigilance, as post-marketing drug safety monitoring, is to identify serious adverse events that can go unnoticed until long after drugs have been approved for marketing. The field of international pharmacovigilance as well as VigiBase were developed after the thalidomide tragedy in the late 1950s (Shankar, 2016), aiming to be complementary to clinical trials, which are built to evaluate drug efficacy more than drug safety (Montastruc *et al.*, 2011). The first case–non-case study was conducted in the field of drug safety during pregnancy in the 1980s: a possible relation between valproic acid and spina bifida was investigated after a cluster of case reports in France from a birth defect registry (Robert and Rosa, 1983). Further studies eventually confirmed the teratogenicity of valproic acid and other antiepileptic drugs (Tomson *et al.*, 2019).

Dydrogesterone (6-dehydro-retroprogesterone) is a non-androgenic man-made derivative of progesterone, being metabolized in hydroxy-derivatives (Schindler, 2009). Unlike other progestins, dydrogesterone and its metabolites have selective progestational activity, do not inhibit ovulation and have no clinically relevant androgenic, estrogenic, or mineralocorticoid activity (Schindler, 2009). Its oral formulation, designed for better resisting enzymatic inactivation in the liver to preserve clinical efficacy, is an important asset compared to micronized vaginal progesterone (Colombo *et al.*, 2006). Since the Lotus I and Lotus II studies, dydrogesterone is widely used in ART for luteal support (Tournaye *et al.*, 2017; Griesinger *et al.*, 2018). It is also commonly used and prescribed in certain regions without clear medical indication, as soon as the pregnancy has been established and until the 12th gestational week in an attempt to prevent miscarriage (Zaqout *et al.*, 2015; Katalinic *et al.*, 2022; Li *et al.*, 2024).

Previous findings regarding the safety of dydrogesterone during pregnancy showed a low rate of spontaneous reporting of birth defects (Queisser-Luft, 2009). No specific pattern of birth defect could be identified and the author stated that a teratogenic effect of dydrogesterone was unlikely (Queisser-Luft, 2009). However, the number of cases analyzed was very limited to ascertain the innocuity of dydrogesterone. Then in 2015, a retrospective case–control study on 202 children born with CHD identified a significant association with dydrogesterone exposure in their mother during pregnancy, highlighting a specific role of dydrogesterone (Zaqout *et al.*, 2015). The timing of exposure during the critical period (i.e. first trimester of pregnancy) raises the possibility of a causal association (Zaqout *et al.*, 2015). More recently, two meta-analyses in 2022 and 2024 considering both observational and experimental studies found no increased risk associated with dydrogesterone (RR = 0.9, 95% CI: 0.6–1.6) (Katalinic *et al.*, 2022, 2024). However, another team estimated that the latter was not robust enough to conclude on the safety of dydrogesterone use, namely on the causal assessment of congenital anomalies (Quadros *et al.*, 2024). Finally, in 2024, a study from China, based on a national cohort of more than 100 000 pregnant women, including about 12% of women exposed to dydrogesterone in early pregnancy, identified a significant association with stillbirth and birth defects (Li *et al.*, 2024). Interestingly, no association was found for progesterone in that study, which was the second most consumed drug in the cohort.

The association between exogenous progestins and teratogenicity, especially CHD or hypospadias, is a longstanding debate in the literature (Levy *et al.*, 1973; Greenberg *et al.*, 1977; Wiseman and Dodds-Smith, 1984; Carmichael *et al.*, 2005). A positive association between CHD and sex hormones was first described in 1973 (Levy *et al.*, 1973), then corroborated by other studies (Nora *et al.*, 1978; Hadjigeorgiou *et al.*, 1982), including the United States Collaborative Perinatal Project (Heinonen *et al.*, 1977). Our study design, based on worldwide reporting, allowed us to assess a differential birth defect reporting between dydrogesterone and progesterone, yielding in a possible safety signal regarding dydrogesterone use during pregnancy. Although the teratogenic mechanisms of dydrogesterone are unknown, several hypotheses are worth discussing. Since progestins are prescribed for their anti-abortifacient effects during early pregnancy, it was hypothesized that they might increase the survival of embryos with CHD (Hook, 1994). However, studies have shown that progestin administration for preventing miscarriage does not systematically result in a significantly higher incidence of live births (Siew *et al.*, 2018; Coomarasamy *et al.*, 2019; Devall *et al.*, 2021). Another mechanism frequently producing birth defects associated with medication exposure is endocrine disruption (van Gelder *et al.*, 2010). Dydrogesterone is a man-made progesterone derivative with a favorable pharmacokinetic profile compared to progesterone, including a higher bioavailability allowing use by oral route. It is rapidly absorbed and completely metabolized (van Amsterdam *et al.*, 1980): after oral administration, maximum plasma levels of dydrogesterone and its main metabolite 20-dihydrodydrogesterone are achieved in <3 h (Schindler, 2009). Dydrogesterone has a more potent and more specific activity via the progesterone receptor than natural progesterone, showing no affinity for androgenic, estrogenic, glucocorticoid, or mineralocorticoid receptors (Schindler *et al.*, 2008; Schindler, 2009). Endogenous progesterone is a major precursor of testosterone in the testicular steroidogenesis that is induced by hCG in the male embryo. Testosterone is responsible for most of the masculinization process, either directly or indirectly, after being converted into dihydrotestosterone (DHT) by the 5α reductase. During pregnancy, fetal androgens arise from testicular steroidogenesis under the action of placental hCG, from the placental steroidogenesis and, to a lesser extent, from the adrenal steroidogenesis. However, studies have shown that male development is more susceptible to endocrine disruption than female development (Sharpe, 2006), such that isolated cases of hypospadias may result either from a defective production of androgens, more specifically DHT, or from a defective action of the androgens on their receptor (Aarskog, 1971). It has also been proposed that compromised androgen production or action by exposure to exogenous progestins during early pregnancy might result in hypospadias in the offspring (Carmichael *et al.*, 2005). Therefore, hypothesizing that dydrogesterone may alter any of these pathways, this could lead to a decrease in the production or action of androgens, triggering isolated cases of hypospadias.

### Strengths and limitations

Our study has strengths and limitations. First, covering more than 90% of the world’s population from about 150 countries, VigiBase provides an advantageous frame to analyze rare adverse events, including adverse pregnancy outcomes at a global scale in real-world settings (Contejean *et al.*, 2023). Hence, disproportionality analyses allow the identification of potential safety signals, based on only a few exposed cases. In this study, we used the ROR as disproportionality estimate, which provides sensitive findings compared to other methods (Jiao *et al.*, 2024). Their ability to provide early signal detection make necessary the conduct of confirmatory studies, although disproportionality estimates have been shown, in certain circumstances, to be well correlated with the strength of the risk association (Grundmark *et al.*, 2014). Nevertheless, while similar in concept, ROR is not intended to be compared with odds-ratio in case–control studies, as they are not risk estimators but estimators of the risk of reporting, which might be influenced by external factors. Significantly, increased ROR supports an association between the drug and the adverse event, but whether it is causal or not requires validation in other studies, including other sources of data. However, this approach has been largely used and validated to identify new adverse drug reactions, notably in pregnant women (Sessa *et al.*, 2019; Chouchana *et al.*, 2020; Desaunay *et al.*, 2024). Furthermore, randomized clinical trials are not designed to assess rare adverse events, such as major birth defects. In the present case of dydrogesterone, we evidenced a safety signal regarding urogenital birth defects. Those defects might sometimes be diagnosed during the first year of life and not at birth. Hence, a follow-up after birth is required to ensure their diagnosis, which is not the case even in randomized clinical trials being set up to assess dydrogesterone efficacy for preventing miscarriage in the field of ART.

Second, spontaneous reporting is associated with under-reporting, inherent to pharmacovigilance systems, impeding the measurement of the actual incidence of birth defects in the study cohort (Montastruc *et al.*, 2011). Although under-reporting is theoretically not likely to be differential between study groups and alter the findings, we acknowledge that some factors may influence reporting, such as local policies, communications, or media attention for example.

Third, drug causality, not being the same for all cases, is challenging particularly for events such as adverse pregnancy outcomes. Additionally, exact dose and timing of drug exposure during pregnancy is not often reported, as great variability exists in the availability of information and its clinical relevance depending on the country of reporting and the type of reporter, undermining precise assessment of drug causality in the reported birth defects.

Lastly, confounding factors, including indication bias, may be present; indeed, studies have shown a significantly increased risk of birth defects in children conceived by ART compared to naturally conceived children (Wen *et al.*, 2012), due to factors associated with the ART procedures overall, but also to factors such as the underlying infertility, as well as a long time-frame for conception (Zhu *et al.*, 2007). Environmental and behavioral factors are also not measured. However, taking into account these possible biases in this study, we performed a case-by-case review to ascertain the reporting of the birth defect and its classification according to EUROCAT.

Foremost, our analyses showed robust results, being consistent through sensitivity analyses and the comparison with ART drugs. In particular, progesterone, being an active comparator, allows mitigation of indication bias, as well as improving the performance of the disproportionality estimator, with a better ability to detect true positives. Finally, our results strengthen the safety signal also identified in the recent maternal drug exposure cohort from China (Li *et al.*, 2024).

## Conclusion

To summarize, despite intrinsic limitations, this observational analysis, based on real-world data, showed a large, and consistent across analyses, increase in reporting of birth defects, mainly CHD and isolated hypospadias, in pregnancies exposed to dydrogesterone, especially when comparing to progesterone. Along with other studies, this possible safety signal emphasizes the need for further investigation regarding the fetal safety profile of dydrogesterone.

## Supplementary Material

hoae072_Supplementary_Data

## Data Availability

Aggregated data are publicly available on https://www.vigiaccess.org/.
