# Correction to: Birth defects reporting and the use of dydrogesterone: a disproportionality analysis from the World Health Organization pharmacovigilance database (VigiBase)

**DOI:** 10.1093/hropen/hoaf029

**Published:** 2025-06-03

This is a correction to: Alexandra Henry, Pietro Santulli, Mathilde Bourdon, Chloé Maignien, Charles Chapron, Jean-Marc Treluyer, Jean Guibourdenche, Laurent Chouchana, Birth defects reporting and the use of dydrogesterone: a disproportionality analysis from the World Health Organization pharmacovigilance database (VigiBase), *Human Reproduction Open*, Volume 2025, Issue 1, 2025, hoae072, https://doi.org/10.1093/hropen/hoae072.

The following information has been added to the Conflict of interest section of the manuscript: M.B., C.C, C.M and P.S have received institutional funding from Merck, Ferring, Theramex, Gedeon Richter, and Besins. M.B. has received personal honoraria from Merck, Ferring, Gedeon Richter, Theramex, IBSA, and Organon for lectures, presentations, or educational events, and has received non-monetary support for attending meetings from Ferring and Gedeon Richter. C.C has received personal honoraria from Merck, Besins, Gedeon Richter, and Theramex, and non-monetary support for attending meetings from Besins, Gedeon Richter, and Merck. He is the Founder and Past-President of the Society for Endometriosis and Uterine Disorders (SEUD), an unpaid role. C.M. has received personal consulting fees from Gedeon Richter and Ferring and honoraria from Merck Serono, Ferring, Besins, IBSA, and Organon for lectures or educational events. She has received non-monetary support for attending meetings from Ferring, Besins, and Gedeon Richter. P.S. has received personal honoraria from Merck, Ferring, Besins, Gedeon Richter, Theramex, IBSA, and General Electrics Medical Systems for lectures and educational events. He has also received non-monetary support for attending meetings from Merck, Ferring, Besins, Gedeon Richter, Theramex, and IBSA. He is a Board Member of SEUD and serves on the Editorial Boards of RBMO and GOF, all unpaid roles. J.G. has received institutional funding from Pregnomic and DiaSorin. He has received personal honoraria from Elsevier and Merck for lectures and educational events. He is a Board Member of ABA and SFE, an unpaid position. L.C., J.-M.T., and A.H. declare no conflicts of interest.

While the authors did not consider the aforementioned activities to be financial or personal relationships that could have influenced the design, conduct, or reporting of this work, the authors wish to add these disclosures in the interest of full transparency (no funding was received for this study). The published article has been updated to include the corrected Conflict of interest section.

In addition, the authors of the above article would like to apologise for (i) a minor discrepancy in data reporting and (ii) an imprecision in the study group names used in the article, which were not spotted previously but could lead to misinterpretation of the overall findings of the study. The authors apologise for these minor oversights and would like to assure readers that they do not affect the overall results, discussion and conclusions presented in the article. The changes are summarised below.

In the extended abstract, the first two sentences of the MAIN RESULTS AND THE ROLE OF CHANCE inaccurately state:

*Study cohort consisted of 362 183 safety reports in pregnant women, among which 50 653 reports were related to the use of drugs for ART, including 145 with dydrogesterone and 1222 with progesterone. Of these, 374 (0.7%) were cases of birth defects: 60 with dydrogesterone and 141 with progesterone, including 48 and 92 cases compatible with major birth defect cases according to EUROCAT classification, respectively.*

It should be:

*Study cohort consisted of 362 183 safety reports in pregnant women, among which 3101 reports were related to the use of drugs for ART, including 145 with dydrogesterone and 1222 with progesterone. Of these, 374 (12.1%) were cases of birth defects: 60 with dydrogesterone and 141 with progesterone, including 48 and 92 cases compatible with major birth defect cases according to EUROCAT classification, respectively.*

In the first paragraph of the ‘Cases description’ section of the ‘Results’, the imprecision in the number of safety reports related to the use of drugs for ART (and thus, the percentage of cases of birth defects) was again quoted:

*The study cohort consisted of 362 183 safety reports in pregnant women, among which 50 653 reports were related to the use of drugs for ART, including 145 with dydrogesterone and 1222 with progesterone (Supplementary Fig. S1, Supplementary Table S2). Of these, query retrieved 470 possible cases of birth defects in reports with ART drugs, including 374 (0.7%) that were retained as cases of birth defects after case-by-case review: 60 birth defects cases were reported with dydrogesterone and 141 with progesterone.*

This should be (with a clarification added regarding the number of individual case safety reports related to ART drugs, including uses for non-pregnancy related conditions):

*The study cohort consisted of 362 183 safety reports in pregnant women, among which 3101 safety reports were related to the use of drugs for ART (of 50 653 reports overall with these drugs regardless of the indication), including 145 with dydrogesterone and 1222 with progesterone (Supplementary Fig. S1, Supplementary Table S2). Of these, query retrieved 470 possible cases of birth defects in reports with ART drugs, including 374 (12.1%) that were retained as cases of birth defects after case-by-case review: 60 birth defects cases were reported with dydrogesterone and 141 with progesterone.*

The wording of the flow-chart of the study (Supplementary Figure S1) has been similarly modified; this is provided in an updated version of the Supplementary data file.

Finally, in Table 3, study group names in the primary and secondary analysis sections have also been clarified:

**Table 3.** Reporting and odds ratios of reporting of birth defects with dydrogesterone users within the WHO global safety database

	Birth defect cases	Non cases	ROR (95% CI)
**Primary analysis**			
Dydrogesterone users	60	85	5.4 [3.9-7.5]
Any drug users	41,991	320,192	Ref
**Secondary analysis**			
Dydrogesterone users	60	85	6.0 [4.2-8.5]
Any other ART drug users	314	2,642	Ref
Head-to-head comparison*			
Dydrogesterone users	60	85	5.4 [3.7-7.9]
Progesterone users	141	1,081	Ref

ROR, reporting odds ratio

* For detailed characteristics of all individual case safety reports suspected to be related to dydrogesterone or progesterone, see Supplementary Table S2

**Primary analysis** category: ‘Any other drug users’ has been updated to ‘Any drug users’. **Secondary analysis** category: ‘Any ART drug users’ has been updated to ‘Any other ART drug users’

The study group name ‘ART drug users’ has also been correspondingly updated to ‘Any other ART drug users‘ in Supplementary Table S3 which is also provided in the updated Supplementary data file.

## Supplementary data

Supplementary data are available at *Human Reproduction Open* online.

## Supplementary Material

hoaf029_Supplementary_Data

