# Supplementary material for: Birth defects reporting and the use of dydrogesterone: a disproportionality analysis from the World Health Organization pharmacovigilance database (VigiBase)
Source: Hum Reprod Open. 2025 Jan 2;2025(1):hoae072. doi: 10.1093/hropen/hoae072 (PMC11726828; doi:10.1093/hropen/hoae072)
Supplement: hoae072_Supplementary_Data [file hoae072_supplementary_data.docx]

**Supplementary Figure S1. Flowchart of the study**

**
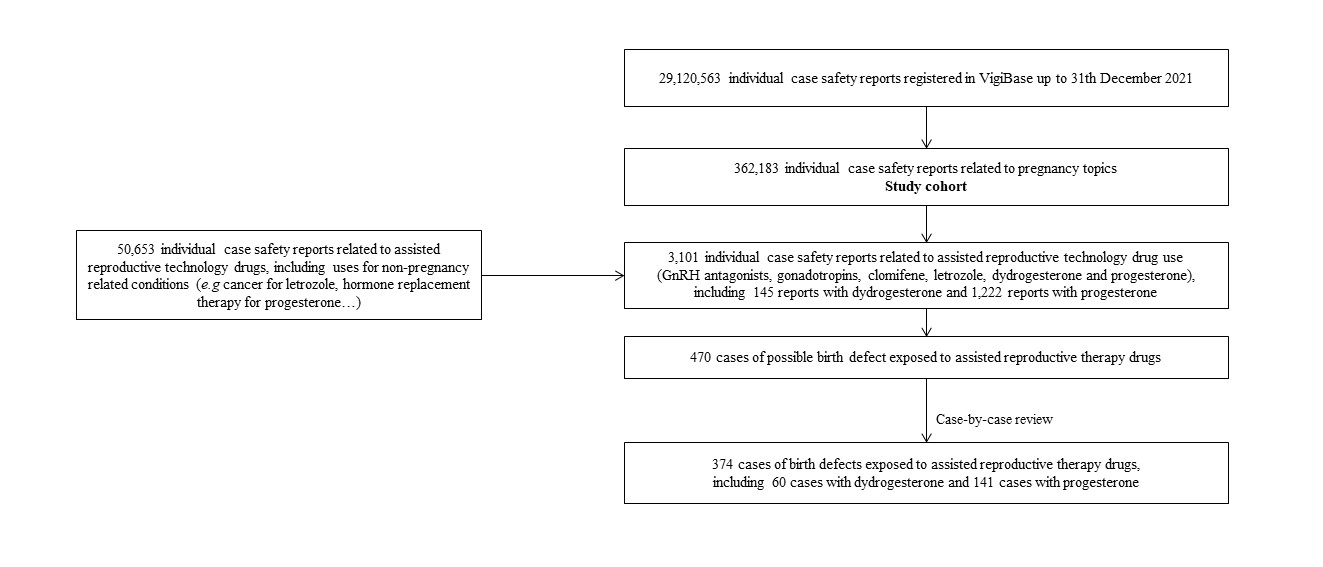
**

**Supplementary Table S1: List of teratogenic drugs**

| **Teratogenic drugs** | |
| --- | --- |
| **Antineoplasic and Immunomodulatory drugs** | fingolimod (L04AA27), leflunomide (L04AA13), lenalidomide (L04AX04), methotrexate (L04AX03, L01BA01), mycophenolic acid (L04AA06), thalidomide (L04AX02), teriflunomide (L04AA31) |
| **Retinoids for systemic use** | acitretin (for psoriasis treatment) (D05BB02), alitretinoin agents for dermatitis (D11AH04), etretinate (D05BB01), isotretinoin for systemic use (D10BA01), tretinoin (L01XX14) |
| **Retinoids for topical use** | adapalene (D10AD03, D10AD53), alitretinoin antineoplastic agent (L01XF02), isotretinoin (D10AD04), tifarotene (D10AD06), tretinoin (D10AD01, D10AD51) |
| **Antiepileptic drugs** | carbamazepine (N03AF01), fosphenytoin (N03AB05), oxcarbazepine (N03AF02), phenytoin (N03AB52), topiramate (N03AX11), valproic acid (N03AG01 without 3400934876233, 3400935444271, 3400934876691) |
| **Drugs for affective disorders** | valpromide (N03AG02), lithium (N05AN01), divalproate (3400934876233, 3400935444271, 3400934876691) |
| **Antithyroid preparations** | carbimazole (H03BB01), thiamazole (H03BB02) |
| **Vitamin K antagonists** | acenocoumarol (B01AA07), fluindione (3400933484132, 3400931183280, 3400931183112), warfarin (B01AA03) |
| **HMG CoA reductase inhibitors** | atorvastatin (C10AA05, C10BA06, C10BX03, C10BX06, C10BX11, C10BX15), fluvastatin (C10AA04), lovastatin (C10AA02), pitavastatin (C10AA08), pravastatin (C10AA03, C10BX02), rosuvastatin (C10AA07, C10BA06), simvastatin (C10AA01, C10BA02, C10BA04) |
| **Other drugs acting as teratogens** | acetazolamide, antiglaucoma preparation (S01EC01) |

**Supplementary Table S2. Characteristics of all individual case safety reports suspected to be related to dydrogesterone or progesterone**

| **Reporting characteristics** | **Dydrogesterone (n=145)** | **Progesterone**  **(n=1222)** |
| --- | --- | --- |
| **Continent of reporting**  Europe  North America  Asia  Oceania  Africa  Latin America | 99 (68%)  2 (1%)  38 (26%)  1 (1%)  4 (3%)  1 (1%) | 417 (34%)  573 (47%)  178 (15%)  13 (1%)  9 (1%)  32 (3%) |
| **Type of reporter**  Physician  Pharmacist  Other Health professional  Consumer  Unknown | 90 (62%)  8 (6%)  7 (5%)  27 (19%)  13 (9%) | 448 (37%)  40 (3%)  139 (11%)  353 (29%)  242 (20%) |
| **Reporting year**  Before year 1980  Years 1980 to 1990  Years 1991 to 2000  Years 2001 to 2010  Years 2011 to 2020  Year 2021 | 8 (6%)  7 (5%)  3 (2%)  11 (8%)  91 (63%)  25 (17%) | 3 (0%)  75 (6%)  92 (8%)  159 (13%)  763 (62%)  130 (11%) |
| **Number of reports by disorder categories***  Congenital, familial and genetic disorders  Foetal disorders  Lactation related topics  Neonatal disorders  Normal pregnancy conditions and outcomes  Pregnancy, labor and delivery complications  Termination of pregnancy and risk of abortion | 68  2  1  6  9  88  32 | 181  68  10  133  173  683  400 |

Data are presented as N (%)

***** As each report may include several disorders, number of reports by categories is superior to total number of reports.

**Supplementary Table S3. Odds ratios of reporting of birth defects in dydrogesterone users within the WHO global safety database (sensitivity analyses)**

| **Sensitivity analysis restricted to years 2001 to 2020** | | | |
| --- | --- | --- | --- |
|  | **Birth defect cases** | **Non cases** | **ROR (95% CI)** |
| ***Primary*** | | | |
| Dydrogesterone users | 37 | 65 | **4.1 [2.7-6.1]** |
| Any other drug users | 34,503 | 245,876 | Ref |
| ***Secondary*** | | | |
| Dydrogesterone users | 37 | 65 | **4.3 [2.8-6.6]** |
| Any other ART drug users | 256 | 1,941 | Ref |
| ***Head-to-head comparison*** | | | |
| Dydrogesterone users | 37 | 65 | **3.7 [2.3-5.7]** |
| Progesterone users | 124 | 798 | Ref |
|  | | | |
| **Sensitivity analysis restricted to reports by healthcare professionals** | | | |
|  | **Birth defect cases** | **Non cases** | **ROR (95% CI)** |
| ***Primary*** | | | |
| Dydrogesterone users | 50 | 55 | **5.9 [4.0-8.7]** |
| Any other drug users | 25,013 | 162,678 | Ref |
| ***Secondary*** | | | |
| Dydrogesterone users | 50 | 55 | **4.9 [3.3-7.4]** |
| Any other ART drug users | 256 | 1,383 | Ref |
| ***Head-to-head comparison*** | | | |
| Dydrogesterone users | 50 | 55 | **4.4 [2.9-6.9]** |
| Progesterone users | 107 | 522 | Ref |

WHO, world health organization; ROR, reporting odds-ratio
